# Validity and Reliability of Elbow Range of Motion Measurements Using Digital Photographs, Movies, and a Goniometry Smartphone Application

**DOI:** 10.1155/2018/7906875

**Published:** 2018-11-29

**Authors:** Renée Keijsers, Elisa L. Zwerus, Dagmar R. M. van Lith, Koen L. M. Koenraadt, Pjotr Goossens, Bertram The, Michel P. J. van den Bekerom, Denise Eygendaal

**Affiliations:** ^1^Department of Orthopaedic Surgery, Amphia Hospital, Breda, Netherlands; ^2^Department of Orthopaedic Surgery, Amsterdam UMC, Netherlands; ^3^Shoulder and Elbow Unit, Department of Orthopaedic Surgery, Onze Lieve Vrouwe Gasthuis, Amsterdam, Netherlands; ^4^Department of Physiotherapy, Amphia Hospital, Breda, Netherlands; ^5^Foundation for Orthopaedic Research, Care and Education, Amphia Hospital, Breda, Netherlands

## Abstract

**Introduction:**

Range of motion (ROM) is closely monitored before and after surgery for stiff elbow and during rehabilitation. Measurements in the home environment may be helpful to increase involvement and adherence of the patient. Therefore, our objective is to investigate the validity and inter- and intraobserver reliability of 3 alternative methods to assess the ROM by the patient in a home-based situation, in comparison to the universal goniometer (UG). We hypothesize that all 3 alternative methods will be valid alternatives and show a level of reliability equivalent to UG.

**Methods:**

Goniometric measurements of elbow flexion, extension, pronation and supination using photography, movie, and a smartphone application were obtained. The validity of these measurement methods was compared to UG. The interobserver and intraobserver reliability were calculated for all measurement methods.

**Results:**

Photography and movie based goniometry of the elbow showed good validity in flexion and extension. The interobserver and intraobserver reliability were found to be good to excellent for photo and movie but moderate to poor for UG and the smartphone application.

**Conclusions:**

Photo or movie based goniometry seems to be a useful option for initial and follow-up measurement of the elbow ROM, both in the outpatient clinic and in a home environment. Based on our study, the smartphone application we used is not recommended.

## 1. Introduction

Reliable measurement of the range of motion (ROM) of the elbow is important for both the initial assessment and at follow-up, to assess the results of surgery or to monitor rehabilitation. Using one reliable and reproducible measurement method between healthcare professionals and the patient himself is beneficial to monitor the effect of different interventions.

In general, the ROM is assessed by an examiner by visual estimation or the use of a universal goniometer (UG) [1]. Amongst the possible alternative measurement methods are for example photography, movie, or a smartphone based application [2–7]. Photography and movie based measurements have several advantages compared to UG. For example, it provides a permanent image, which can be used to visually demonstrate the improvement to the patient, possibly helping to improve patient adherence. This relationship has been proved in other medical fields, for example, in measurements of blood pressure in the home environment. In literature a rise of almost 10% in medicine compliance and significant blood-pressure reduction has been described [8, 9]. However, for those measurement methods a transfer from the camera or mobile phone to a computer with measurement software is necessary. Also, it requires an assisting person to take the picture or movie.

Smartphone based applications, based either on photography or an accelerometer, may be less time consuming and easy to use in a home environment. Measuring at home gives the patient the possibility to take more responsibility for his rehabilitation and update the health care provider between appointments.

Previous studies showed that both visual estimation and UG measurement have a good to excellent inter- and intraobserver reliability [1, 10–21]. In literature on several other joints, excellent reliability for photography and smartphone apps was observed. Studies include photography of the elbow [2, 21] or knee [4] and smartphone apps based on an accelerometer principle for the knee [3, 5, 6] or shoulder [7].

For the elbow specifically, it is unclear which of the available goniometric measurement methods is the most reliable to measure flexion, extension, pronation, and supination. Therefore, the objective of the current study is to investigate the validity and reliability of photography, movie, and smartphone application based goniometry compared to UG. We hypothesize that all 3 alternative methods to measure elbow ROM will show a similar level of accuracy and inter- and intraobserver reliability, equivalent to UG.

## 2. Materials & Methods

### 2.1. Study Design

In this study measurements of the ROM of the elbow were collected using the UG, a smartphone application, photography, and movies. The pictures and the movies itself were made by independent volunteering ‘photographers,' in general a family member who accompanies the participant. UG measurements were conducted by two health care professionals: a resident in the orthopedic department and a physiotherapist. The smartphone application measurements were done by the patient, with the aforementioned two health care professionals reporting the outcomes. Ethical approval was waived by the local ethical committee.

### 2.2. Study Population

Subjects with or without elbow pathology, accompanied by a volunteer able to handle a digital camera (‘photographer'), were included. Both must be 18 years or older, have sufficient knowledge of the Dutch language and physically, and cognitively able to perform the proceedings in the measurement protocol. Subjects were recruited in a general hospital, a sports and performance center and physiotherapy clinic. These locations and flexible inclusion criteria were chosen to ensure adequate representation of a population with and without elbow complaints.

### 2.3. Study Procedure

Demographic data on height, weight, age, gender, and hand dominance were collected for each subject. In all subjects the active ROM of the elbow (dominant side) was measured three times with UG and three times by an application on a smartphone, by both observers independently. The order of the method of measurement by the two observers (UG vs application and examiner 1 vs examiner 2) was randomized by means of block randomization (blocks of 4). The photos were taken twice and the movie once by the ‘photographer' in stated order, after each block of UG and smartphone app sessions.

Subjects were instructed to carry out the four positions of the ROM: maximum flexion, extension and functional forearm rotation in pro- and supination. Attention must be paid to the difference between functional forearm rotation and pronation and supination. The functional forearm rotation measures the motion of forearm rotation in the two radioulnar joints (proximal and distal), combined with carpal rotation. Pronation and supination measure only the motion of the two radioulnair joints and are therefore a few degrees smaller. A study by Cimatti et al. showed that both methods could be used in clinical practice with excellent reliability [22]. In this study it is decided to use the functional forearm rotation because it is easier to implement for laymen. This means that in our results supination stands for forearm rotation in supination direction and pronation for forearm rotation in pronation direction.

#### 2.3.1. Universal Goniometer

Two observers measured all subjects' ROM three times independently with an UG. Between measurements of the two observers in one subject, a minimal interval of 5 minutes was applied. A stainless-steel goniometer was used and measurements were blinded for the observer by reversing the goniometer. Measurements were recorded with accuracy of 1 degree. A predefined protocol was used by both observers, based on recommendations in previous literature by using bony landmarks [14, 19, 21, 23–29].

For flexion and extension measurements of the elbow, the shoulder was held in 90 degrees of forward flexion with the forearm maximally supinated. The acromion and radial styloid process were landmarks for the goniometers' arms and the lateral epicondyle as the center of rotation. Supination and pronation were measured with a neutral position of the shoulder (0° shoulder abduction) and 90° of elbow flexion and a pencil placed over the distal palmar groove of the hand. The center of rotation for pronation and supination was over the head of the third metacarpal and the goniometers' arms were placed parallel to the humeral midline and parallel to the pencil.

#### 2.3.2. Photography

**Figure 1:**
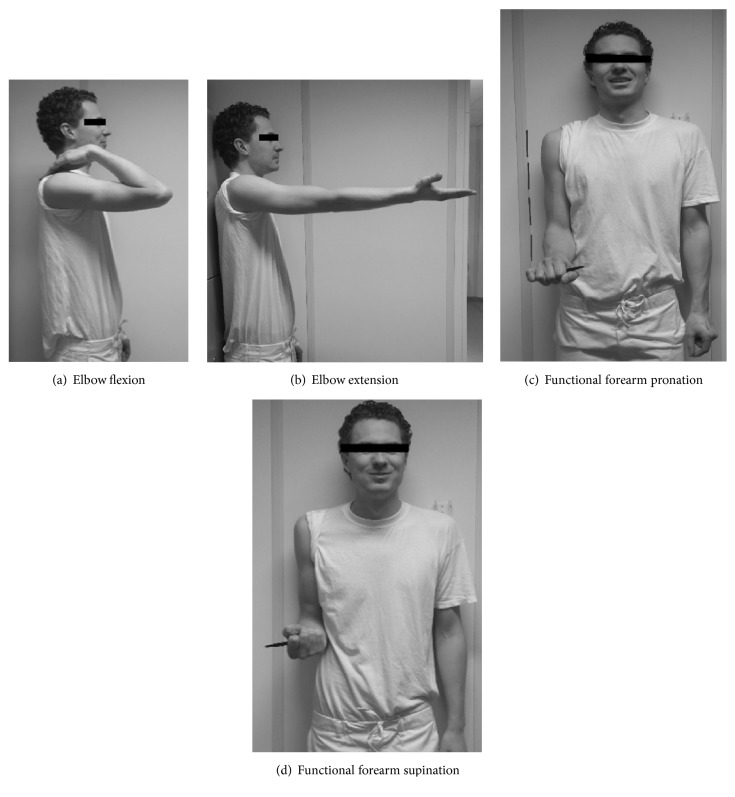


The photographers were instructed how to take the photos by a comprehensive and simplified protocol with sample pictures (Figures 1(a)–1(d)). The positions and motions were standardized as for the UG measurements described in the previous paragraph. The ‘photographer' takes two series photos of the subject with a minimal interval of 20 minutes using a digital camera. In total 8 pictures were taken by each photographer. Elbow ROM on pictures was measured two times by both observers separately with a minimal interval of one day, using Kinovea software (Version 0.8.15, open source project, www.kinovea.org).

#### 2.3.3. Movie

The movie was made by the photographer using the same protocol and device as for the photo as described in the previous paragraph. Subjects were instructed to slowly (in 5 seconds) carry out the movements from maximum flexion to maximum extension (movie 1) and from maximum supination to maximum pronation (movie 2). In total two movies, one for flexion-extension and one for pronation-supination movement, were taken by each photographer. Elbow ROM on the same movie was measured twice with a minimal interval of one day by each of two aforementioned observers separately using Kinovea software.

#### 2.3.4. Smartphone Application

The Joint Goniometry application (version 2.1, Diomidis Papas via App Store) for smartphones was used in simple mode for the elbow ROM measurements. This app is based on the principle of an accelerometer, comparable to other accelerometer based smartphone applications available in the App Store and Google Play. All 4 movements, as mentioned in de previous paragraphs, were measured three times by the two aforementioned observers. The previously mentioned landmarks were used as for the UG. When the subjects arm was held in the right position, the smartphone was placed with the middle of the bottom on the center of rotation and aligned to the proximal arm. The correct position was confirmed by a tap on the screen, followed by alignment on the distal arm and again confirmed by another tap. Measurements were blinded using a nontransparent elastic band on the screen.

### 2.4. Data and Statistical Analysis

In the study preparation phase, the sample size was calculated. Based on a significance of 0.05 (alpha) and power of 0.20 (beta), assuming a moderate correlation for our four measurement modalities, at least 18 participants were required.

All data were analyzed using SPSS version 22 (Armonk, NY, USA: IBM Corp) and Medcalc (version 16.1). A p value of <0.05 was considered statistically significant. Data was checked manually for outliers in distribution. Subject characteristics are presented using descriptive statistics and 95% confidence intervals (CI).

Photography, movie and smartphone application based goniometry were individually compared to the UG measurements to analyze validity. The mean of the measurements in all three methods (photo, movie, and app) of both observers was compared to the mean of the measurements with the UG. The agreement between the alternative measurement methods and the UG was calculated using the intra-class correlation coefficient (ICC). Because the ICC uses variance between subjects' ROM measurements to calculate reliability, a large variation between subjects will lead to a higher ICC. This could possibly draw a misleading conclusion of good reliability [30, 31]. Therefore we decided in our study to provide the mean difference (∆) and accompanying 95% confidence intervals (CI) as well. To calculate these values, the mean of all three goniometer measurements (for maximum flexion, extension, pro-, and supination separately) was compared to the mean of all three measurements by photo, movie, or app.

For the interobserver reliability, the same photo (photo 2) and movie were measured by both observers. For the smartphone application and UG the second measurement of both observers were compared. In the same way, the mean difference (∆) and 95% CI were determined; the means of the measurements of observer 1 were compared to the means of observer 2. The intraobserver reliability was determined based on the measurements of the first observer (resident in the orthopedic department). The measurements of photo 1 were compared to photo 2. For the smartphone application and the UG the measurements of all three moments were compared. For the movie two measurement moments of the same movie were compared. Again, ∆ and SD were determined. The interobserver and intraobserver reliability were calculated using ICC.

For both validity and reliability analysis, the ICCs were calculated using a two-way random effects model where both people effects and measures effects are random. ICC between 0.75 and 1.00 indicates excellent reliability, between 0.60 and 0.74 good, between 0.40 and 0.59 moderate and ICC of ≤0.40 indicates a poor reliability [32]. Bland-Altman plots defining the limits of agreement (LOA) were used to determine whether a good correlation between two measurement methods also means that there is a good agreement between two methods [33]. A t-test was subsequently conducted to check for systematic errors. In addition, the linear regression was examined to check for proportional errors.

## 3. Results

### 3.1. Subject Demographics

The study included 40 subjects (21 males and 19 females), each accompanied by an inexperienced ‘photographer.' One subject had an elbow disorder without a functional disability. The mean age was 48 years (95% CI 43-54), mean height 175 centimeters (95% CI 172-177), and mean weight 83 kilograms (95% CI 78-89). Four subjects (10%) were left-handed and 36 right-handed.

### 3.2. Validity of Measurement Methods

For flexion and extension, both photography and movie based measurements show a good to excellent correlation with UG. In pronation and supination measurement using photography and movie showed a moderate correlation with UG. The correlation between photography and movie measurements was good. The smartphone application correlated good with the UG in pronation and supination. Poor correlation for the smartphone application was shown for extension measurement, while flexion showed a moderate correlation. A proportional error was observed for extension in both photo and movie. This means that, with increasing angles, the difference in angle between photo and movie with UG increased. The validity for the photography-, movie-, and smartphone application based measurement methods compared to UG, the ∆ and 95% confidence intervals (CI) was reported in Table 1.

### 3.3. Interobserver and Intraobserver Reliability Analysis

The interobserver reliability was excellent for photography and movie based measurements (Table 2). Results for the smartphone application and UG were moderate to good. The mean differences between observers' measurements are in all cases less than 5 degrees; however the accompanying 95% CI shows a very wide range for UG and the smartphone application.

Intraobserver reliability was good to excellent for photography based measurements and excellent for movie (Table 3). The intraobserver reliability was poor to moderate for the smartphone application and moderate to excellent for UG. The mean differences between to measurements of the same observer are under 5 degrees, apart from the pronation measurement using the smartphone.

## 4. Discussion

The current study reported validity and interobserver and intraobserver reliability for universal goniometry compared to 3 alternative measurement methods for elbow goniometry including photography, movie, and a smartphone application. Validity appeared to be dependent on which elbow motion was measured. Photography and movie based goniometry showed better validity in flexion and extension, whereas the smartphone application showed better validity for pronation and supination. With respect to the reliability, interobserver and intraobserver reliability were found to be good to excellent for photo and movie but were predominantly poor to moderate for UG and the smartphone application. This means that in our study the variance in measurements amongst and within the observers is smaller for photo and movie compared to UG and the smartphone application.

In our study a systematic (proportional) error underestimating the extension measurement was observed by both photo and movie when compared to UG. Therefore, the results of extension from photo and movie are not interchangeable with UG. These findings are in line with previous literature on elbow and knee goniometry [2, 3, 21]. It is questionable if this error is caused by the photo, movie, or UG measurement. Difficulties identifying the rotation center landmark has been designated as source for an error in the extension using photography or UG. Hence, it seems that in literature the UG is underestimating the extension angle [2, 4, 21].

With respect to the reliability of the UG measurements, our study results are only partially in line with previous literature. Literature on interobserver and intraobserver reliability shows ICC values within a wide range, from 0.45-0.99, yet most ICCs were over 0.70 [11–16, 19]. In our study, the interobserver reliability of UG was moderate to good, ranging from 0.40 to 0.71 and the intraobserver reliability was moderate to excellent, ranging from 0.47 to 0.84. The wide range for reliability in both the literature and our study could be explained by the fact that the observers only had a few years' experience.

The reliability of photography in our study is in line with previous studies, however for the smartphone application our study demonstrated lower reliability. In literature, for both photography and smartphone apps excellent reliability was observed for several joints. Studies include photography of the elbow [2, 21] or knee [4] and smartphone apps based on an accelerometer principle for the knee [3, 5, 6] or shoulder [7]. All studies showed that photography or a smartphone application offer better reliability and are less dependent on the observers' experience compared to UG. A possible explanation for the disappointing results for the smartphone application in our study is the use by laymen. When tapping the screen, the application sometimes faltered and deviating results were not always recognized by the subjects.

We did not find literature using movie based goniometry. The excellent ICCs we found for the movie could be an overestimation, because two observations of measurements by each observer were based on a single movie.

Consideration should be given to the fact that UG might not be the most reliable method for elbow ROM measurement, especially in inexperienced examiners, as shown both in the literature and by the current study [10, 21]. Also, goniometry is used on a moving subject, unlike photography and movie, where measurements are carried out on a still image. Furthermore, measurements of functional forearm rotation (thus including carpal rotation) and pronation and supination are frequently placed under a common denominator. However, this accounts for all types of measurement methods we used.

This study is not without limitations. Subjects under 18 years old were excluded because of legal issues in younger patients. Moreover, in our study sample of 40 participants no patients with functional disabilities were included. Our results may not be automatically generalized for a population with elbow pathology without additional research. However, previous literatures comparing the reliabilities of goniometric elbow measurements of pronation and supination show good inter- and intrarater reliability for noninjured and even better for injured subjects [17, 22].

To verify correctness of measurements, measurements took place on our location, still simulating the home environment. It appeared that some participants required minimal adjustments to conduct the protocol correctly; in particular, during the imaging of the maximum supination to maximum pronation some of the participants forgot to keep the elbow against the body. For measurements in the home environment it is recommended to emphasize this in the protocol and, for example, practice the measurements with the patients the first time at the outpatient clinic or rehabilitation/physiotherapy center. It also might be illustrative to provide an accompanying instruction film when the measurements will actually take place in a home situation.

In order to obtain a measurement as reliable as possible, we recommend to use photography or movie for measurements both at the outpatient clinic and in the home environment. This allows the clinician to save the photo or movie and demonstrate the change (e.g., before and after intervention or follow-up) by showing sequential photos or movies to the patient. This provides the opportunity to increase patient engagement and adherence to rehabilitation therapy. Furthermore, between therapy sessions and for the long term follow-up the patient has to visit the clinic less frequently without losing important information on the patients' progress.

## 5. Conclusion

Based on this study, we recommend the use of photo or movie based goniometry for flexion and extension measurements of the elbow motion. These methods can be used in both the clinic and a home environment to increase the amount of follow-up moments and patient engagement during the rehabilitation process.

## Figures and Tables

**Table 1:** Validity of different measurement methods compared to UG measurements (ICC) and mean difference (∆) and 95% confidence intervals (CI) (in degrees).

UG vs.		*Photography*	*Movie*	*Smartphone application*
*Flexion*	ICC (95% CI) ∆ (95% CI) (°)	0.71 (0.51-0.83) 0 (-1.9 to 1.9)	0.63 (0.41-0.79) 0 (-1.9 to 1.9)	0.57 (0.32-0.75) 0 (-2.2 to 2.2)

*Extension*	ICC (95% CI) ∆ (95% CI) (°)	0.76 (0.58-0.87) 1 (-0.2 to 2.2)	0.78 (0.63-0.88) 0 (-1.2 to 1.2)	0.28 (-0.05-0.55) 5 (3.8 to 6.2)

*Pronation*	ICC (95% CI) ∆ (95% CI) (°)	0.44 (0.15-0.66) 4 (1.5 to 6.5)	0.45 (0.17-0.67) 2 (-0.5 to 4.5)	0.67 (0.47-0.82) 1 (-0.5 to 2.5)

*Supination*	ICC (95% CI) ∆ (95% CI) (°)	0.50 (0.23-0.70) 2 (-0.8 to 4.8)	0.47 (0.18-0.68) 1 (-2.4 to 4.4)	0.61 (0.37-0.77) 1 (-1.5 to 3.5)

**Table 2:** Interobserver reliability (ICC) and mean difference (∆) and 95% CI (in degrees) of UG, photography, and movie and smartphone application measurements.

		*UG*	*Photography*	*Movie*	*Smartphone application*
*Flexion*	ICC (95% CI) ∆ (95% CI) (°)	0.41 (0.07-0.65) 5 (2.8 to 7.2)	0.83 (0.65-0.92) 1 (0.1 to 1.9)	0.86 (0.75-0.92) 1 (0.1 to 1.9)	0.66 (0.45-0.81) 2 (-0.5 to 4.5)

*Extension*	ICC (95% CI) ∆ (95% CI) (°)	0.65 (0.43-0.80) 1 (-0.5 to 2.5)	0.93 (0.88-0.96) 0 (-0.9 to 0.9)	0.88 (0.77-0.93) 1 (-0.2 to 2.2)	0.56 (0.31-0.74) 2 (-0.2 to 4.2)

*Pronation*	ICC (95% CI) ∆ (95% CI) (°)	0.40 (0.11-0.63) 2 (-1.4 to 5.4)	0.90 (0.76-0.95) 3 (1.5 to 4.5)	0.82 (0.56-0.91) 3 (1.5 to 4.5)	0.55 (0.29-0.73) 3 (-1.3 to 7.3)

*Supination*	ICC (95% CI) ∆ (95% CI) (°)	0.71 (0.51-0.83) 0 (-2.2 to 2.2)	0.89 (0.77-0.94) 2 (0.5 to 3.5)	0.96 (0.93-0.98) 1 (0.1 to1.9)	0.48 (0.20-0.67) 1 (-2.7 to 4.7)

**Table 3:** Intraobserver reliability (ICC) and mean difference (∆) and 95% CI of UG, photography, and movie and smartphone application measurements.

		*UG*	*Photography*	*Movie*	*Smartphone* *application*
*Flexion*	ICC (95% CI) ∆ (95% CI) (°)	0.50 (0.31-0.67) 3 (2.1 to 3.9)	0.87 (0.81-0.92) 1 (0.7 to 1.3)	0.94(0.89-0.97) 1 (0.7 to 1.3)	0.60 (0.36-0.76) 4 (3.4 to 4.6)

*Extension*	ICC (95% CI) ∆ (95% CI) (°)	0.84 (0.75-0.91) 2 (1.7 to 2.3)	0.82 (0.73-0.88) 1 (0.7 to 1.3)	0.96(0.93-0.98) 1 (0.7 to 1.3)	0.45 (0.16-0.66) 3 (2.4 to 3.6)

*Pronation*	ICC (95% CI) ∆ (95% CI) (°)	0.71 (0.57-0.81) 3 (2.4 to 3.6)	0.72 (0.59-0.81) 3 (2.1 to 3.9)	0.94(0.98-0.97) 2 (1.7 to 2.3)	0.58 (0.33-0.75) 6 (3.2 to 8.8)

*Supination*	ICC (95% CI) ∆ (95% CI) (°)	0.47 (0.28-0.65) 4 (2.1 to 5.9)	0.71 (0.57-0.81) 3 (2.4 to 3.6)	0.95(0.92-0.98) 2 (1.7 to 2.3)	0.31 (0.02-0.56) 5 (3.1 to 6.9)

## Data Availability

Data is available on request with the corresponding author.

## Abbreviations

ROM: Range of motion
UG: Universal goniometer
CI: Confidence interval
ICC: Intraclass coefficient.
